# Peritoneal Infusion of Oxygen Microbubbles Alters the Metabolomic Profile of the Lung and Spleen in Acute Hypoxic Exposure

**DOI:** 10.3390/bioengineering11080761

**Published:** 2024-07-27

**Authors:** Christina Lisk, Alex Fan, Francesca I. Cendali, Kenta Kakiuchi, Delaney Swindle, David I. Pak, Robert Tolson, Abby Grier, Keely Buesing, Seth Zaeske, Angelo D’Alessandro, Mark A. Borden, David C. Irwin

**Affiliations:** 1 Cardiovascular and Pulmonary Research Laboratory, Department of Medicine, University of Colorado Denver—Anschutz Medical Campus, Denver, CO 80204, USA; christina.lisk@cuanschutz.edu (C.L.); delaney.swindle@cuanschutz.edu (D.S.); david.i.pak@cuanschutz.edu (D.I.P.); robert.tolson@cuanschutz.edu (R.T.); seth.zaeske@co.rvu.edu (S.Z.); 2 Biomedical Engineering Program, University of Colorado, Boulder, CO 80309, USA; alexander.fan@colorado.edu (A.F.); kenta.kakiuchi@colorado.edu (K.K.); mark.borden@colorado.edu (M.A.B.); 3 Department of Biochemistry & Molecular Genetics, Graduate School, University of Colorado, Anschutz Medical Campus, Aurora, CO 80045, USA; francesca.cendali@cuanschutz.edu (F.I.C.); abby.grier@unmc.edu (A.G.); angelo.dalessandro@cuanschutz.edu (A.D.); 4 Department of Surgery, Division of Acute Care Surgery, University of Nebraska Medical Center, Omaha, NE 68198, USA; keely.buesing@unmc.edu; 5 Department of Mechanical Engineering, University of Colorado, Boulder, CO 80309, USA

**Keywords:** Metabolomics, OMBs, spleen, lung, hypoxia

## Abstract

Administration of oxygen microbubbles (OMBs) has been shown to increase oxygen and decrease carbon dioxide in systemic circulation, as well as reduce lung inflammation and promote survival in preclinical models of hypoxia caused by lung injury. However, their impact on microenvironmental oxygenation remains unexplored. Herein, we investigated the effects of intraperitoneal administration of OMBs in anesthetized rats exposed to hypoxic ventilation (FiO_2_ = 0.14). Blood oxygenation and hemodynamics were evaluated over a 2 h time frame, and then organ and tissue samples were collected for hypoxic and metabolic analyses. Data showed that OMBs improved blood SaO_2_ (~14%) and alleviated tissue hypoxia within the microenvironment of the kidney and intestine at 2 h of hypoxia. Metabolomic analysis revealed OMBs induced metabolic differences in the cecum, liver, kidney, heart, red blood cells and plasma. Within the spleen and lung, principal component analysis showed a metabolic phenotype more comparable to the normoxic group than the hypoxic group. In the spleen, this shift was characterized by reduced levels of fatty acids and 2-hydroxygluterate, alongside increased expression of antioxidant enzymes such as glutathione and hypoxanthine. Interestingly, there was also a shuttle effect within the metabolism of the spleen from the tricarboxylic acid cycle to the glycolysis and pentose phosphate pathways. In the lung, metabolomic analysis revealed upregulation of phosphatidylethanolamine and phosphatidylcholine synthesis, indicating a potential indirect mechanism through which OMB administration may improve lung surfactant secretion and prevent alveolar collapse. In addition, cell-protective purine salvage was increased within the lung. In summary, oxygenation with intraperitoneal OMBs improves systemic blood and local tissue oxygenation, thereby shifting metabolomic profiles of the lung and spleen toward a healthier normoxic state.

## 1. Introduction

Hypoxia and/or hypercapnia may arise from a multitude of underlying conditions, such as acute respiratory distress syndrome [1,2,3], chronic obstructive pulmonary disease [4,5], asthma exacerbation [6] and high-altitude pulmonary edema [7,8]. Hypoxia is associated with an increase in reactive oxygen species (ROS) [9,10], such as superoxide [11], as well as inflammation [12] and apoptosis of a variety of cell types [13,14]. Acute hypoxic exposures have correspondingly been shown to alter metabolism [15], which is the focus of the present investigation.

Oxygen microbubbles (OMBs) are 1–10 µm diameter lipid-coated oxygen gas microparticles designed to alleviate hypoxia by delivering oxygen and hypercapnia by removing carbon dioxide owing to their biocompatibility, aqueous physicochemical properties and high surface-to-volume ratio [16]. Kheir et al. demonstrated that intravenous OMB injections promote survival in a rabbit-resuscitation model [17], but rapid injection of a large OMB volume needed to treat severe or prolonged hypoxia may lead to deleterious hemodynamic effects [18]. Research into treating systemic hypoxia, therefore, shifted to OMB administration into body cavities, such as the peritoneal cavity [19], thoracic cavity [20] and colon [2]. In 2014, Feshitan et al. demonstrated the effectiveness of peritoneal oxygenation with OMBs in increasing peripheral blood saturation (SpO_2_) and increasing survival in a rat pneumothorax model [19]. Subsequent studies showed the effectiveness of intraperitoneal OMBs in a rabbit tracheal occlusion model [21], rat lipopolysaccharide (LPS) model [22], rat smoke inhalation (SI) model [23] and pig SI model [2]. Administration to the colon has also been demonstrated to increase safety and feasibility by replacing the need for surgical port placement with more clinically practical enema delivery [2]. Together, these results emphasize the efficiency of systemic oxygen delivery and carbon dioxide removal by OMBs in both small and large animal models of hypoxia. Interestingly, intraperitoneal and colonic administration of OMBs has also been shown to reduce lung inflammation and promote lung healing [2,19,22], even though the lung is not in direct contact. The lung-protective mechanism of OMBs is not yet well understood.

Metabolomic analysis offers advantages by lacking animal-specificity and, therefore, can closely align physiological states across species compared to other methods [24]. Since 2017, its utilization has significantly expanded across various research domains [25]. Furthermore, by assessing multiple organs, it facilitates a comprehensive evaluation of intervention effects on the entire body, despite tissue-level analysis [26,27]. For example, Cheng et al. found similar alterations in metabolite levels in both the brains and livers of mice both before and after an acute hypoxic exposition [27]. Metabolomic analysis allows investigators to utilize well-established metabolic pathways from various animals and tissues to determine the effects of therapeutics. In this study, we aimed to determine if peritoneal infusion of OMBs alters the metabolomic profile in an acute hypoxic rodent model. While numerous studies have explored blood oxygenation with OMB administration, few studies have evaluated the effects at the tissue level, and none have evaluated the effects on organ metabolism. We therefore employed metabolomics on multiple organs as a primary outcome to examine the effectiveness of OMB administration.

## 2. Methods

### 2.1. Oxygen Microbubble (OMB) Preparation

Lipid solution was prepared at a concentration of 10 mg/mL with a 9:1 molar ratio of DSPC: DSPE-PEG2000 (Avanti Polar Lipids, Alabaster, AL, USA). OMBs were prepared in a scaled-up manufacturing process as previously described [19]. Briefly, lipid solution was transferred to a 500 mL syringe and pumped into a custom-built continuous flow system. The enclosed system is oxygen-saturated and circulates lipid solution through a sonication chamber where OMBs are generated. OMBs accumulate in a jacketed, oxygen-saturated cooling column. OMBs were subsequently collected from this column in 60 mL syringes and concentrated by centrifugation, achieving a final gas volume fraction of ~70%. The size distribution and microscopic images of OMBs were obtained using an optical particle analyzer (Accusizer 780A: Pss Nicomp 92821, Brea, CA, USA) and an inverted microscope (BX-52: Olympus, Evident, Tokyo, Japan).

### 2.2. Hypoxia Model and Treatment by Peritoneal Infusion of OMBs

**Figure 1:**
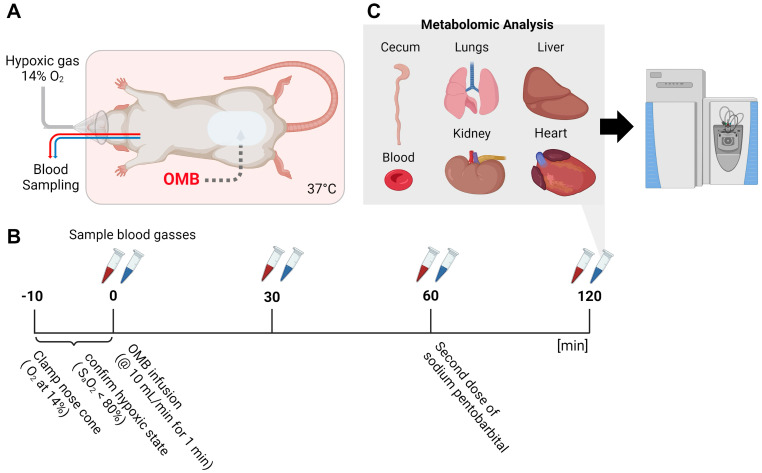
Experimental Design. (**A**) Rats were anesthetized, an infusion tube was inserted into the peritoneal cavity for OMB infusion, and both the jugular vein and right carotid artery were catheterized to obtain venous and arterial blood. Hypoxic (HX) and hypoxic-treated-with-OMBs (HX + OMB) study animals were then exposed to FiO_2_ = 14% via a nose tube. (**B**) 10 min prior to the first blood draw (both arterial and venous), hypoxic animals were confirmed to be hypoxic using an i-STAT handheld blood analyzer with C4 + blood gas cartridges. Once the animal was confirmed to be hypoxic (or normoxic, NX), blood was isolated from the jugular vein and right carotid artery (time = 0, baseline). Blood was then removed from both arterial and venous lines every 30 min for a total of 2 h. (**C**) Cecum, lungs, liver, blood (plasma and red blood cells) and the heart were all analyzed for metabolomics.

All animal experiments were evaluated and approved by the University of Colorado Anschutz Medical Campus Institutional Animal Care and Use Committee (IACUC). Twelve-week-old, male Sprague Dawley rats weighing 350 to 400 g were used for these experiments. Prior to exposing the rats to hypoxic conditions, the animals were weighed, anesthetized with ketamine and xylazine, and placed onto a warming pad for the remainder of the study. Next, an abdominal incision was made to access the peritoneal cavity, and the peritoneal infusion tubing was secured with a purse string suture. The left jugular vein and right carotid artery were then catharized to obtain venous and arterial blood gas measurements (Figure 1A).

The rats were randomly assigned to 3 treatment groups: normoxic control (NX), hypoxic control (HX) and hypoxic OMB-treated (HX + OMB), with n = 3–5 replicates collected for each group. For the HX and HX + OMB conditions, the anesthetized rat was placed on a nose cone, and fraction of inspired oxygen (FiO_2_) was reduced to 14% via an electronic flow meter. Veinous and arterial blood were sampled every 15 min, and blood gases were measured using an i-STAT handheld blood analyzer with C4 + blood gas cartridges (Abbott Life Sciences, Chicago, IL 60064, USA). In a subset of rats (n = 3/per group), a 60 mg/kg dose of pimonidazole HCl (Hypoxyprobe, NPI Inc., Burlington, MA 01803, USA) diluted in saline was administered via the jugular vein catheter 30 min prior to the study endpoint. OMB-treated rats subsequently received a 100 mL/kg dose of OMBs (concentration ~1010 OMBs/mL) infused via a syringe pump at a rate of 10 mL/min while warming the animal to 37 °C. Hypoxic control rats did not receive an OMB infusion. Subsequently, venous and arterial blood gases were measured at 30 min, 1 h and 2 h, as shown in Figure 1B. NX rats were not placed on the restricted oxygen nose cone but instead were allowed to breathe the ambient air for the duration of the 2 h time course. At 120 min post-infusion, sodium pentobarbital (Fatal-Plus, Vortech Pharmaceuticals, Dearborn, MI 48126, USA) was delivered to the animal to initiate euthanasia; the animal was immediately exsanguinated via the carotid artery, and the acquired blood was put into a 10 mL EDTA tube on ice. Next, blood was spun down at 1000 G to avoid red blood cell (RBC) hemolysis and separated for RBCs and plasma samples. Immediately following exsanguination, OMBs were removed from the peritoneal cavity to determine their size distribution and concentration. Samples of 10 mg of heart, lungs, kidneys, cecum and liver were immediately harvested and snap-frozen in liquid nitrogen for metabolomics characterization via mass spectrometry (Figure 1C). In a separate group of animals, HX and HX + OMB rats (n = 3) organs were harvested for fixation and antibody staining using the FITC-Mab1 supplied with the Hypoxyprobe kit.

### 2.3. Metabolomic Analysis

Blood was processed using 10 µL of sample (diluted 1:10). Tissues were pulverized with a mortar and pestle in a liquid nitrogen bath, then extracted at 15 mg/mL with cold MeOH:MeCN:H_2_O (5:3:2, *v*:*v*:*v*). Suspensions were vortexed vigorously for 30 min at 4 °C. Insoluble material was pelleted by centrifugation (18,213× *g*, 10 min, 4 °C), and supernatants were isolated for analysis on a Thermo Vanquish UHPLC coupled to a Thermo Q Exactive MS, as previously described [28].

### 2.4. Statistical Analysis

**Figure 2:**
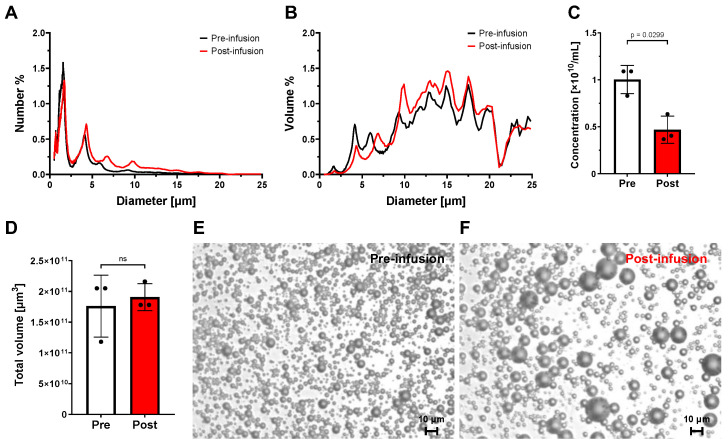
Changes in Intraperitoneal OMBs. (**A**) Number-weighted size distribution of pre- (black line) and post-infusion (red line). (**B**) Volume-weighted size distribution of pre- (black line) and post-infusion (red line). (**C**) Concentration of OMBs from pre- (gray bar) and post-infusion (red bar). (**D**) Total volume isolated from pre- (gray bar) and post-infusion (red bar). (**E**) Microscopic images of OMBs pre-infusion. (**F**) Microscopic images of OMBs pre-infusion. Scale bars are 10 µm.

**Figure 3:**
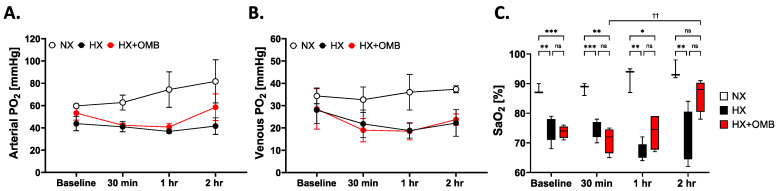
OMBs Influence Arterial PO_2_ and Oxygen Saturation. (**A**) Arterial partial pressure of oxygen from normoxic (NX, white circles), hypoxic (HX, black circles) and hypoxic-treated-with-OMBs (HX + OMBs, red circles) at each time point. (**B**) Venous partial pressure of oxygen (PO_2_) from NX (white circles), HX (black circles) and HX + OMBs (red circles) at each time point. (**C**) Arterial blood oxygen saturation (SaO_2_) from NX (white bars), HX (black bars) and HX + OMBs (red bars). * *p* < 0.05, **,^††^ *p* < 0.01, *** *p* < 0.001, ns = not significant.

Data are presented as a mean ± standard deviation (SD). Comparisons between two groups in Figure 2 were performed with the paired *t*-test. Comparisons between groups in Figure 3 were performed with the Tukey’s multiple comparison test following the 2-way analysis of variance (ANOVA). Statistical analysis was completed using the statistical software package GraphPad Prism (version 10.2.1). Multivariate analyses, including hierarchical clustering analysis (HCA), principal component analysis (PCA) and partial least squares discriminant analysis (PLS-DA) were performed with MetaboAnalyst 6.0 [29]. Statistical analysis is embedded into the MetaboAnalyst program. Data were scaled via auto-scaling, which uses the mean-centered for each metabolite divided by the standard deviation of each variable. Significance was defined as *p* ≤ 0.05 within MetaboAnalyst. Pathway analysis and network analyses were performed via Omicsnet 2.0 [30].

## 3. Results

### 3.1. Morphological Changes in Intraperitoneal OMBs

Figure 2A,B, respectively, depict the number- and volume-weighted size distributions of pre- (black line) and post-infusion (red line). These results show that the percentage population of the larger (5–15 μm diameter) bubbles increased, and smaller (<2 μm diameter) bubbles decreased in the retrieved OMB dispersion. The concentration of OMBs exhibited a significant decrease (Figure 2C), while the total volume remained unchanged (Figure 2D), suggesting coalescence and/or Ostwald ripening of OMBs within the peritoneal cavity. The same alterations were confirmed in microscopic images (Figure 2E,F). Prior work in our group has shown that these OMBs in storage for several days do not experience such a dynamic change in their size and concentration. Such morphological changes are consistent with mechanisms of gas exchange [31]. Most importantly, the shift in microbubble size and concentration indicated that, while in the peritoneal cavity, the OMBs exchanged their oxygen gas core for nitrogen and carbon dioxide. 

### 3.2. OMBs Influence Arterial PO_2_ and Oxygen Saturation 

Peritoneal administration of OMBs in lung injury models was previously shown to increase systemic oxygen and decrease systemic carbon dioxide, presumably by oxygenating hemoglobin within red blood cells (RBCs) as they transit through the peritoneal cavity and nearby vascular beds, feeding into the splanchnic circulation [2,19,22,23]. However, it was unclear if a systemic oxygenation effect would be observed in the healthy lung subjected to acute hypoxia, where equilibration of the RBCs as they transit the pulmonary vasculature may lead to the release of oxygen into the hypoxic alveoli and thereby dampen the systemic effect. To this end, blood samples were taken at baseline and 30-, 60- and 120-min post-infusion (Figure 1A). At baseline, there was no difference in arterial and venous PO_2_ between the cohorts (Figure 3A,B), although there was a significantly higher arterial blood oxygen saturation (SaO_2_) in NX vs. HX and HX + OMB (Figure 3C). By the 30 min and 1 h time points, the HX and HX + OMB cohorts remained hypoxic from breathing 14% oxygen compared to room air (21% oxygen) in the NX control rats. The arterial PO_2_ (PaO_2_) in the NX group increased ~25% over the study time course as the rats acclimated to the anesthesia (Figure 3A). In contrast, this was not observed in HX-exposed rats, but an increase in PaO_2_ was observed in HX + OMB after 2 h. Figure 3B shows lower venous PO_2_ (PvO_2_) in both HX-exposed cohorts, and we observed no significant differences in changes over time in each group. SaO_2_ values for all groups are shown in Figure 3C. Consistent with PaO_2_ values, hemoglobin arterial oxygen saturation in the HX and HX + OMB cohorts were significantly lower throughout the study time course compared to NX-exposed animals. Supporting the PaO_2_ data, the SaO_2_ values in the HX + OMB group, when compared to the 30-min time point, increased ~14% at 2 h. Also, at 2 h post-infusion, the SaO_2_ values of the HX + OMB rats were not significantly different from the NX control. Not surprisingly, we did not observe any differences in the mean arterial pressure (MAP) or heart rate between groups, demonstrating no acute systemic hemodynamic and cardiovascular toxicity effects of peritoneal OMB administration (Supplemental Figure S1). Taken together, the blood gas data support our hypothesis that OMBs can increase systemic oxygen even in the healthy lungs subjected to hypoxic ventilation, although this systemic effect was not as drastic as in prior lung injury models. For example, a more rapid and substantial increase in systemic oxygenation was observed in pigs following smoke-inhalation injury [2]. To better understand the underlying mechanism, we examined effects on nearby tissue and distally from the peritoneal OMB treatment.

### 3.3. OMBs Show Histological Changes for Attenuating Microenvironment Tissue Hypoxia 

**Figure 4:**
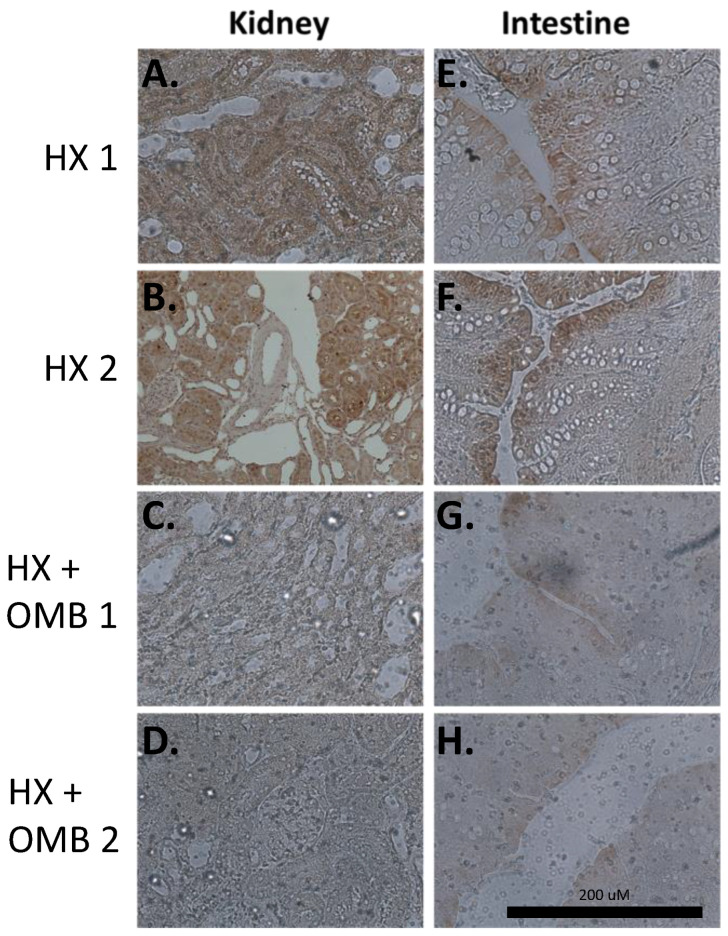
OMBs Alter Tissue Hypoxia in the Kidney and Intestine. Kidneys (**A**–**D**) and cecum (**E**–**H**) isolated from HX rats and HX + OMB rats. A total of 30 min prior to euthanasia, a 60 mg/kg dose of Hypoxyprobe was administered via the jugular vein. The dark brown areas (**A**,**B**,**E**,**F**) emphasize hypoxic microenvironments. OMB treatment decreased the hypoxic microenvironment, as shown in the treated animals (**C**,**D**,**G**,**H**).

To qualitatively confirm OMB oxygenation in the surrounding intraperitoneal organ tissue, we microscopically observed kidneys and intestines for the presence or absence of pimonidazole adducts with a Hypoxic Probe. Pimonidazole adducts are formed when tissue PO_2_ drops below 15 mmHg [32]. Harmonizing with the blood gas data, we observed more adduct (brown staining) in HX compared to HX + OMB tissue with microscopic visualization comparisons (Figure 4), demonstrating that oxygen released from OMB reached the tissue level and attenuated hypoxia within the microenvironment.

### 3.4. OMBs Alter Hypoxic-Induced Changes in Metabolism 

**Figure 5:**
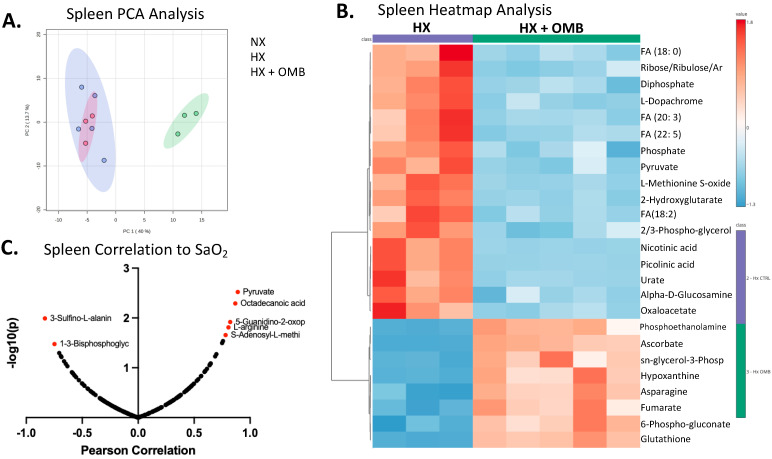
OMBs Alter Hypoxic-Induced Changes in Splenic Metabolism. (**A**) Principal component analysis (PCA) of NX (red), HX (green), HX + OMB (blue). (**B**) Top 25 metabolites via heatmap analysis between HX and HX + OMB. Red squares show an increase in metabolites and blue squares depict a decrease in a specific metabolite. (**C**) Splenic metabolite correlations to SaO_2_.

Organ and cellular metabolisms rapidly change during hypoxic exposure. Thus, we sought to understand how OMB administration altered organ metabolomic profiles in our acute hypoxic rat model. To this end, metabolomic analysis was applied to spleen, liver, kidneys, cecum, lungs, heart, RBCs and plasma. Focusing on the organs that have direct contact with OMBs in the peritoneal cavity (cecum, liver, kidney and spleen), metabolomic analysis via principal cluster analysis (PCA) was used to determine if the HX + OMB group rescued the hypoxic metabolic shift, where only the spleen showed a significant effect (Figure 5A). We then used partial least squares-discriminant analysis (PLS-DA) to determine if the variations between groups in the cecum, liver and kidney could be elucidated, however, HX + OMB groups did not cluster with the NX or HX groups (Supplemental Figure S2). When the spleen from HX + OMB was compared to HX cohorts alone, heatmap visualization showed a metabolic shift away from fatty acid metabolism, decreases in the tricarboxylic acid (TCA) cycle and recovery of antioxidant enzymes in OMB treated rats (Figure 5B). This is evidenced by the distinct down-regulated cluster of fatty acids and 2-hydroxygluterate, and up-regulation of antioxidant enzymes glutathione and hypoxanthine. It is widely recognized that hypoxia promotes fatty acid uptake, synthesis, storage and usage [33,34], production of 2-hydroxgluterate [35] and increased production of reactive oxygen species [10,36].

Because of the direct proximity to OMBs, it is unclear if splenic metabolism was also influenced by changes in RBC hemoglobin oxygen saturation (SaO_2_), as opposed to only oxygen diffusion directly from OMBs in the peritoneal cavity to splenic cells. Thus, to begin to distinguish the hemoglobin oxygen delivery component to spleen metabolomics, a Pearson correlation analysis was undertaken between spleen metabolites and SaO_2_ values. Pearson correlation analysis revealed five up- and two down-regulated metabolites highly correlated to SaO_2_ values (Figure 5C). We interpreted these highly correlated spleen metabolites to SaO_2_ as a reflection of metabolomic changes associated with tissue oxygenation from arterial-phase hemoglobin.

**Figure 6:**
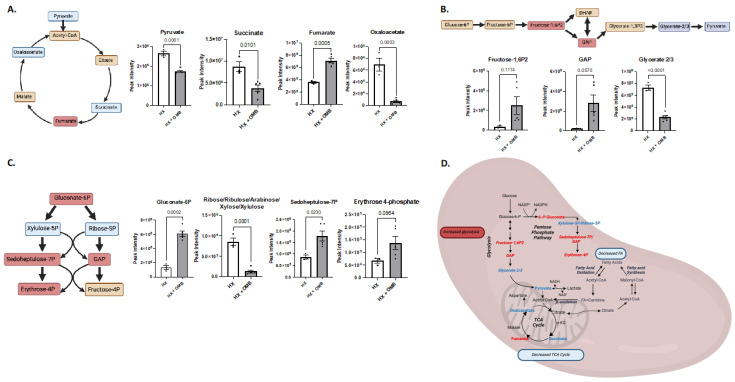
Metabolic Pathways Altered in the Spleen. (**A**) The tricarboxylic acid (TCA) cycle and individual graphs of metabolites within the TCA cycle between HX and HX + OMB. (**B**) The glycolytic pathway and individual metabolites of this pathway between HX and HX + OMB. (**C**) Pentose Phosphate Pathway (PPP) and individual metabolites of this pathway between HX and HX + OMB. (**D**) Overall alteration of multiple metabolic pathways within the spleen after OMB treatment. Downregulated metabolites are highlighted in the blue boxes, while increased metabolites are within the red boxes.

Further analysis of the heatmap emphasized a shuttling effect from the TCA cycle towards glycolysis and the pentose phosphate pathway (PPP). The altered TCA metabolism from HX to HX + OMB is shown in Figure 6A, with decreased metabolites in blue boxes and increased metabolites in red. Here we observed decreases in pyruvate, succinate and oxaloacetate, all of which are key metabolites within the TCA cycle. To understand which metabolic pathway was accelerated in the spleen, we looked at metabolites from both glycolysis and PPP. In Figure 6B, we observed trending increases in frucotse-1,6 bisphosphate (Fuctose-1,6P2) and glyceraldehyde 3-phosphate (GAP), suggesting that the glycolysis pathway was being utilized. Interestingly, glycerate 2/3 decreased significantly. When we investigated metabolites from the PPP, we also observed a significant increase in gluconate-6P, ribose-5P, sedoheptoulose-7P and erythrose 4P. Figure 6D shows the overall altered metabolism.

While differences in the metabolomic signal in the cecum, liver and kidney were not as strong as the spleen, we noted some differences when comparing HX to HX + OMB. Within the cecum, heatmap analysis showed a trending decrease in the top 25 metabolites (Supplemental Figure S3A). IPA analysis indicated this decrease correlated with metabolism of nucleotides, which is confirmed in the heatmap, with significant differences between ATP, UMP, phosphate and nicotinamide. Heatmap analysis of the liver between HX and HX + OMB showed loose clustering (Supplemental Figure S3B). The downregulated metabolites were analyzed through IPA and were correlated with lipid metabolism. This is emphasized in the heatmap, showing a decrease in α-linolenic acid, eicosapentaenoic acid, octadecenoic acid, linoleic acid and hexadecenoic acid. The upregulated metabolites in the liver were associated with amino acid metabolism via IPA analysis. The kidney analysis showed that most of the top 25 metabolites increased (Supplemental Figure S3C). These metabolites correlated with the TCA by IPA analysis, which is highlighted in the heatmap showing an increase in succinate.

**Figure 7:**
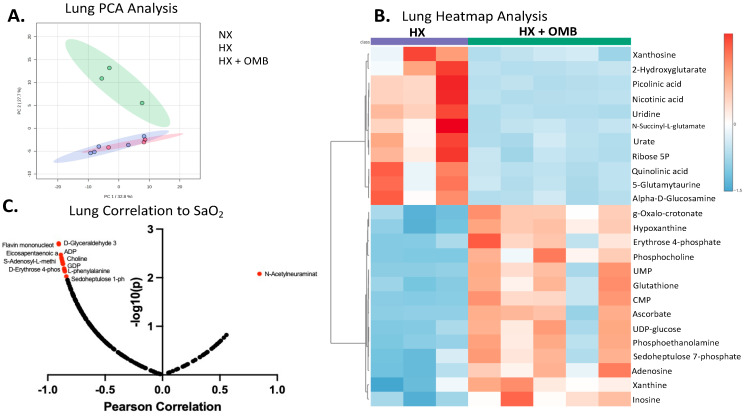
OMBs Alter Hypoxic-Induced Changes in the Lung Metabolism. (**A**) Principal component analysis (PCA) of NX (red), HX (green) and HX + OMB (blue). (**B**) Top 25 metabolites via heatmap analysis between HX and HX + OMB. Red squares show an increase in metabolites and blue squares depict a decrease in a specific metabolite. (**C**) Lung Metabolite Correlations to SaO_2_.

Next, we determined if OMB administration had a broader effect on the cardiovascular system by analyzing the metabolomic profiles of lung and heart tissue. Again, we started with PCA analysis and determined that only the lung had rescued the metabolic phenotype towards NX vs. HX (Figure 7A). The PLS-DA analysis of the heart (Supplemental Figure S4A) was comparable to the cecum, liver and kidney, where HX + OMB was the most differentiated from NX. Interestingly, the PCA of the lung tissue revealed OMB treatment re-establishes a metabolomic profile that more closely resembles a NX metabolism (Figure 7A). Similar to the OMBs’ effect on the spleen metabolomic profile, heatmap analysis showed OMB administration (HX + OMB) also down-regulated 2-hydroxyglutarate and restored antioxidants glutathione and hypoxanthine in the lung when compared to HX alone (Figure 7B). Taken together with PCA analysis, this shows that OMBs positively influenced organs distal to the peritoneal compartment of administration.

Because we hypothesized the OMBs’ influence on lung metabolomics would most likely occur through a mechanism of hemoglobin binding and release, we sought to understand the association in lung metabolism to SaO_2_ values. A Pearson correlation analysis between lung metabolites and SaO_2_ values revealed 10 lung metabolites (nine down- and one up-regulated metabolite) were highly correlated to SaO_2_ (Figure 7C). This indicates that hemoglobin transport of oxygen is important to altering the metabolic phenotype of the lung.

**Figure 8:**
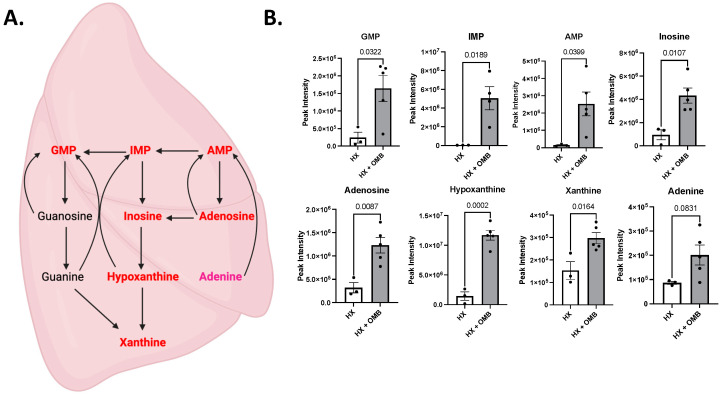
Metabolic Pathways Altered in the Lung. (**A**) Purine metabolism, where OMB treatment increased the metabolites listed in red. (**B**) Individual metabolites which were altered within purine metabolism between HX and HX + OMB.

Deeper analysis of the metabolites within the lung showed significant differences in purine metabolism, specifically the purine salvage pathway. Figure 8A depicts purine metabolism, with the right-hand side of the pathway describing purine salvage. Metabolites shown in red were significantly increased in HX + OMB, compared to HX. Metabolites in pink showed a strong trend for increases but did not reach significance (*p* = 0.0831). Figure 8B shows individual graphs comparing metabolites within the purine salvage pathway. Surprisingly, all metabolites were significantly increased in the HX + OMB groups, apart from adenine, which trended towards a significant increase. Purine salvage is energetically favorable for reproduction of ATP, ADP and AMP, the energy sources for most cells [37]. Recent studies have shown a protective mechanism by purine salvage in pulmonary hypertension [38] and small-cell lung cancer [39].

While the metabolomic signal in the heart was not as strong as in the lung, we noted a trend in decreased metabolites within the top 25. These metabolites were analyzed by IPA and connected with glycolysis because of the decrease in D-Glucose, UDP-GlcNAc and 2,3-DPG (Supplemental Figure S4B). It was recently shown that acute hypoxia increases glycolysis within the heart, which leads to heart failure [40]. Therefore, it is interesting that peritoneal OMBs decreased glycolysis in the heart. 

Finally, PCA on RBCs and plasma did not show strong separation between NX, HX and HX + OMB (Supplemental Figure S5A,B). Interestingly, heatmap analysis of the RBCs between HX and HX + OMB showed two clusters. The increased metabolites were found to be involved with nucleotide metabolism, whereas the decreased metabolites were associated with glutathione conjugation (Supplemental Figure S5C). Comparably, heatmap analysis of the plasma between HX and HX + OMB showed an increased cluster related to amino acid metabolism and a decreased cluster connected to acyl chain remodeling (Supplemental Figure S5D). 

## 4. Discussion

There is a critical need for minimally invasive therapeutics to maintain the oxygenation of vital organs during a hypoxic crisis. Here, we showed improved arterial blood gases, decreased local hypoxia in the kidney and the intestine, and restored organ metabolism to a more normoxic phenotype in rats exposed to 14% hypoxia for 2 h and administered with a peritoneal infusion of OMBs. We observed that multiple metabolic pathways were altered in different organs. The field of metabolomics is a relatively new field, and the direct functional characteristics of these alterations have yet to be fully understood. Together, these data show that peritoneal infusion of OMBs have a beneficial systemic effect of counteracting organ tissue hypoxia and restoring metabolism toward a more normoxic phenotype [41]. 

An acute hypoxic crisis may occur from any health condition that compromises oxygen exchange from the alveolus to hemoglobin within RBCs transiting the pulmonary vasculature, such as smoke inhalation or high-altitude pulmonary edema. In such cases, breathing supplemental oxygen may not be adequate to counter the fall in arterial blood oxygen partial pressure (PaO_2_), increasing the risk of permanent organ damage and death from tissue hypoxia. Our data showed an increase in hemoglobin saturation (SaO_2_), a decrease in pimonidazole adducts in the kidney and intestine, and metabolomic changes in the spleen, liver, cecum, lung and heart, which demonstrates that OMB oxygenation has an effect not only on organs in the peritoneal cavity bathed in OMBs, but also organs distal to the site of administration as well, specifically the lung. 

Because the spleen is a primary lymphoid organ that contains large numbers of immune cells, it may have an outsized role in adverse cardiopulmonary effects during an acute hypoxic crisis. For instance, the spleen has been a therapeutic target for ARDS patients because of its propensity to release immunostimulatory systemic factors, such as cytokines, that adversely affect the lung and other organs [42,43]. Thus, restoring normoxic metabolism to the spleen that reduces local oxidative stress and attenuates the release of inflammatory mediators may be a critical factor to resolve ARDS quickly with improved outcomes. Here, we showed that treatment with OMBs restores antioxidants and increases glutathione conjugation in the spleen during acute hypoxic exposure. Glutathione conjugation is well recognized as a crucial detoxification method for tissue. Glutathione (GSH) is an antioxidant that removes many xenobiotic compounds via conjugation with GSH, allowing for the toxin to be secreted from the cell [44]. Our data therefore suggest that OMBs may increase the rate at which toxins are removed from individual cells with the tissue. 

It is well recognized that alveolar hypoxia increases reactive oxygen species, inflammation and the induction of apoptosis, which all disrupt the efficiency of gas exchange [45,46]. Our data showed that OMBs move the metabolomic profile toward a more normoxic phenotype, suggesting a protective quality via upregulation of glutathione conjugation and antioxidants. Also, atelectasis and alveolar collapse may occur if the production of pulmonary surfactant is disrupted during a hypoxic event. Pulmonary surfactant has been shown to comprise up to 90% phospholipids [47], and herein our data showed that with the peritoneal infusion of OMBs, phospholipid synthesis is increased. This suggests that OMB oxygenation may protect against atelectasis in acute lung injury, preventing or prolonging complete respiratory collapse. 

This is the first preclinical study that evaluates peritoneal administration of OMBs in a rodent model of acute hypoxia exposure and that eliminates confounding factors that occur in more complex lung injury models, such as smoke inhalation or LPS models. Our study provides compelling proof-of-concept, demonstrating that OMBs attenuate the progressive decline of systemic blood gases and improve tissue oxygenation by altering the metabolomic profiles to be closer to those of a healthy, well-oxygenated phenotype. A limitation of this study is that metabolomic-dependent effects of OMBs organ-by-organ were not homogeneous. For example, despite the strong evidence of a positive OMB effect on the spleen and lung metabolism, this shift was not as apparent in liver, cecum, kidney, heart, RBC or plasma. Thus, there are a few discrepancies that warrant deeper investigation. For example, one would have expected a strong metabolic shift toward a NX phenotype in liver, cecum and kidney, as shown in the spleen, because they were also proximal to the intraperitoneal OMBs. These data suggest that not all tissue benefits equally from OMBs’ oxygenation. This warrants further dose-finding and efficacy studies to optimize OMB oxygenation for a more homogeneous response across all organs. Nonetheless, the metabolomic profile of the spleen and lung demonstrates a strong oxygen effect in this acute hypoxic rat model. Moreover, OMBs may be tied to phospholipid synthesis in the lung and protect against alveolar atelectasis. These data suggest a unique therapeutic potential of OMBs for treating an acute hypoxic crisis where patients are at risk for atelectasis, such as smoke inhalation. Like many other proof-of-principle studies testing therapeutic effects before clinical evaluation, our experimental design started treatment at the time of hypoxia exposure instead of determining if the therapeutic effect would reverse the process after a hypoxia already began and had persisted for some time. Therefore, studies determining optimal timing of OMB therapy are warranted.

## 5. Conclusions

In summary, our data showed that intraperitoneal administration of OMBs improved hemoglobin oxygen saturation, resolved tissue hypoxia (as shown by a decrease in pimonidazole adducts in the kidney and intestine) and markedly shifted metabolism in the spleen and lung toward a normoxic state. By incorporating the specific outcomes on arterial oxygen and metabolic profiles, our study demonstrated that rats exposed to hypoxic conditions and treated with OMBs experienced a notable increase in arterial oxygen levels. This was accompanied by a significant metabolic divergence in both the lung and spleen, compared to hypoxic no-treatment controls. Crucially, OMB treatment shifted the metabolic profile in these organs closer to those observed in the normoxia control group, indicating a restoration of metabolic functions toward normal physiological conditions. These findings highlight the robust systemic impact of OMB therapy, not only in enhancing arterial oxygenation but also in normalizing metabolic disturbances caused by hypoxic stress. This dual benefit is especially pertinent in the context of acute hypoxic crises where both oxygen delivery and metabolic integrity are compromised. The improvement in metabolic profiles in critical organs like the lung and spleen further substantiates the potential of OMBs as a therapeutic strategy, offering more than oxygenation, by potentially stabilizing the metabolic functions essential for organ recovery and overall survival. This research highlights the capability of OMB therapy to counter the deleterious effects of tissue and organ hypoxia by both improving oxygenation and realigning metabolism towards a healthier, normoxic phenotype. In conclusion, this proof-of-principle study demonstrates that OMBs could play a crucial role in the clinical management of conditions characterized by acute oxygen deficits, thereby providing a comprehensive approach to restoring organ function and preventing further hypoxic damage. This novel metabolomics approach not only elucidated the efficacy of peritoneal OMB administration, but also demonstrated its utility as an assessment tool, which may be used for other systemic oxygenation technologies, such as peritoneal infusion of oxygenated perfluorocarbons [48].

## Data Availability

The original contributions presented in the study are included in the article/supplementary material, further inquiries can be directed to the corresponding author.

## Supplementary Materials

The following supporting information can be downloaded at: https://www.mdpi.com/article/10.3390/bioengineering11080761/s1, Figure S1: OMB treatment did not alter systemic hemodynamics; Figure S2: Partial Line Least Square Discriminant Analysis (PSL-DA) from OMB Surrounded Organs; Figure S3: Heatmap Analysis from OMB Surrounded Organs; Figure S4: Metabolic Analysis of the Heart; Figure S5: Metabolic Analysis of the Red Blood Cells and Plasma.
