# Supplementary material for: Peritoneal Infusion of Oxygen Microbubbles Alters the Metabolomic Profile of the Lung and Spleen in Acute Hypoxic Exposure
Source: Bioengineering (Basel). 2024 Jul 27;11(8):761. doi: 10.3390/bioengineering11080761 (PMC11352204; doi:10.3390/bioengineering11080761)
Supplement: Supplementary file 1 [file bioengineering-11-00761-s001.zip › bioengineering-3078384-supplementary.pdf]

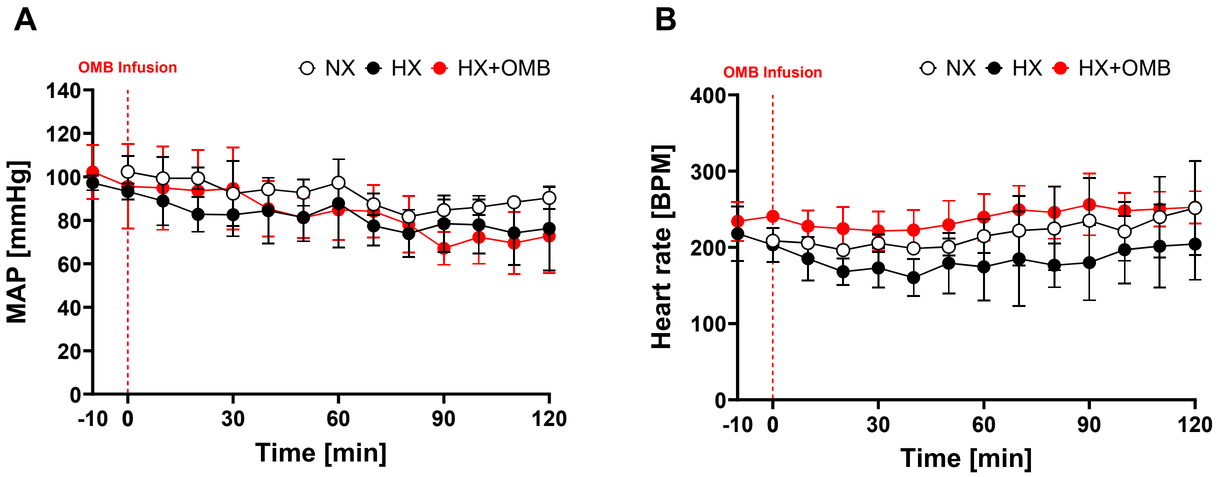

Supplemental Figure S1: *OMB treatment did not alter systemic hemodynamics.* (A) Mean arterial pressure (MAP) and (B) heart rates from normoxic (NX, white circles), hypoxic (HX, black circles), and hypoxic treated with OMBs (HX + OMBs, red circles) at all time points.

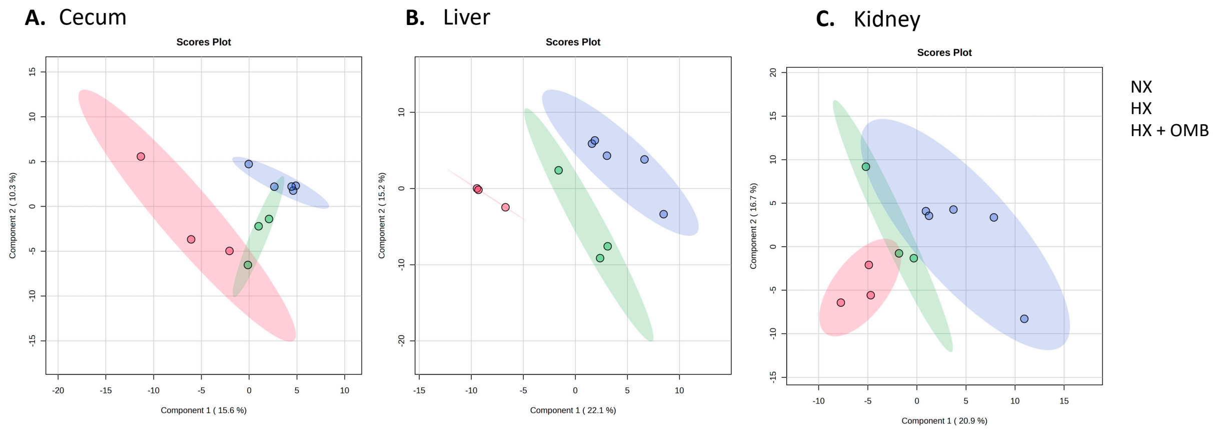

Supplemental Figure S2: *Partial Line Least Square Discriminant Analysis (PSL-DA) from OMB Surrounded Organs.* PSL-DA analysis was done on the (A) cecum, (B) liver, (C) kidney of NX (red), hypoxic (green), hypoxic treated with OMBs (blue) animals.

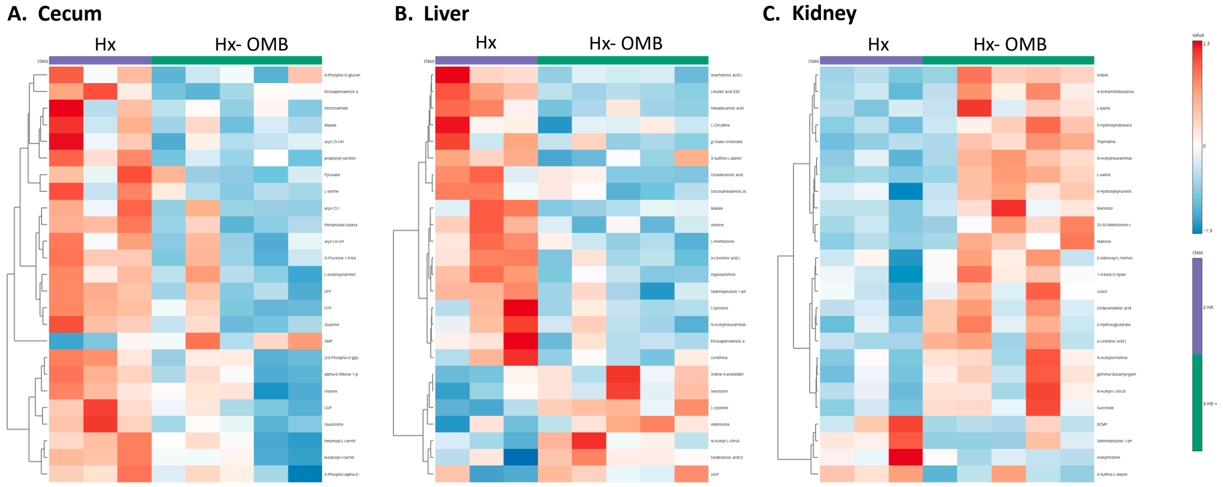

Supplemental Figure S3: *Heatmap Analysis from OMB Surrounded Organs.* Metabolites heatmap analysis for the top 25 from the (A) cecum, (B) liver, (C) kidney.

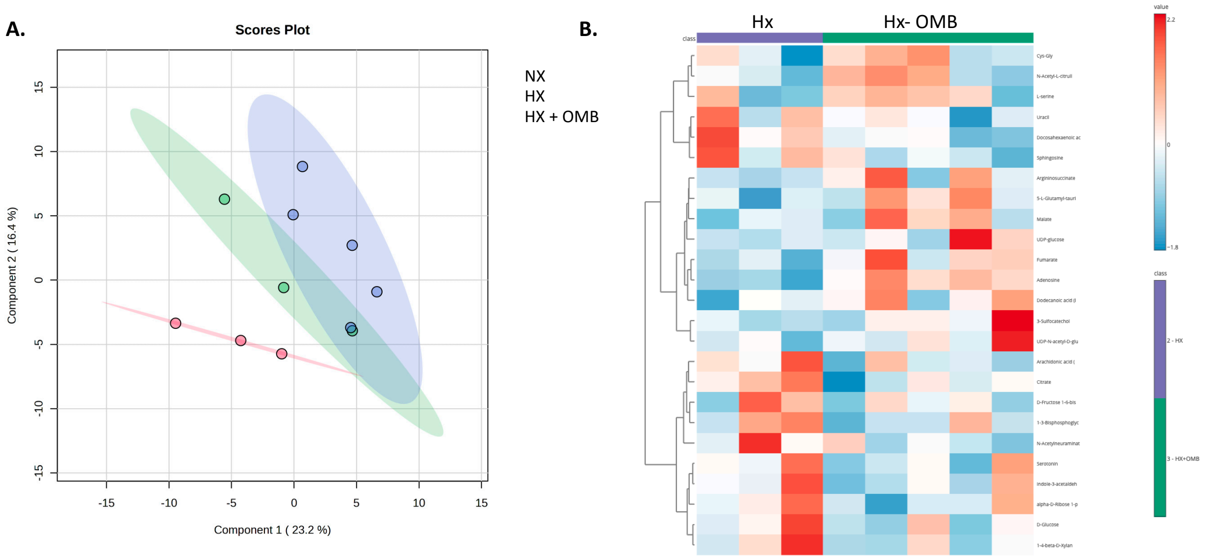

Supplemental Figure S4: *Metabolic Analysis of the Heart.* (A) PSL-DA metabolic analysis between of NX (red), hypoxic (green), hypoxic treated with OMBs (blue) animals. (B) Heatmap analysis of the top 25 metabolites from the whole heart tissue between HX and HX + OMB.

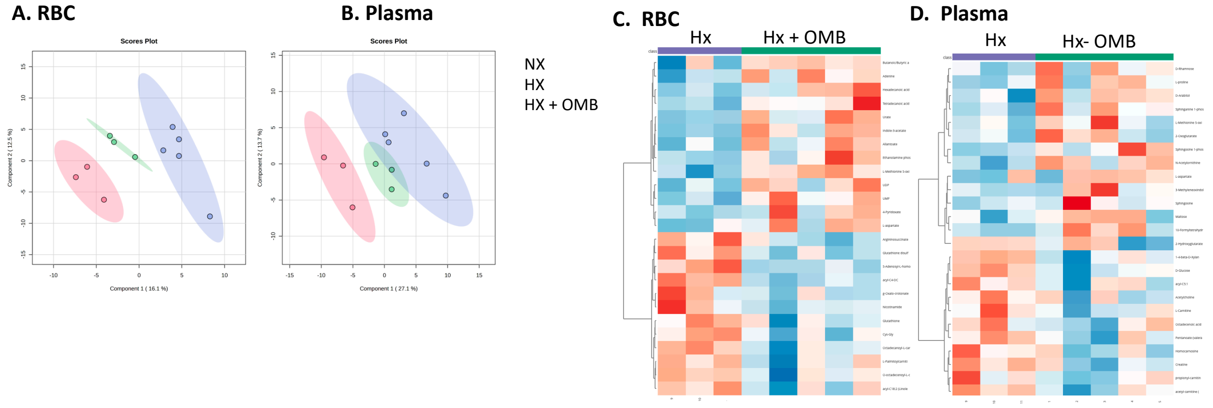

Supplemental Figure S5: *Metabolic Analysis of the Red Blood Cells and Plasma.* (A) RBC PSL-DA metabolic analysis between of NX (red), hypoxic (green), hypoxic treated with OMBs (blue) animals. (B) Plasma PSL-DA metabolic analysis between of NX (red), hypoxic (green), hypoxic treated with OMBs (blue) animals (C) Heatmap analysis of the top 25 metabolites from the RBCs between HX and HX + OMB. (D) Heatmap analysis of the top 25 metabolites from the plasma between HX and HX + OMB.
